# Understanding blast-induced neurotrauma: how far have we come?

**DOI:** 10.2217/cnc-2017-0006

**Published:** 2017-06-08

**Authors:** Ibolja Cernak

**Affiliations:** 1 Faculty of Rehabilitation Medicine, University of Alberta, Corbett Hall 3–48, Edmonton Alberta, T6G 2G4, Canada

**Keywords:** blast-induced neurotrauma, blast injuries, blast traumatic brain injury, clinical research, experimental research, perspectives

## Abstract

Blast injuries, including blast-induced neurotrauma (BINT), are caused by blast waves generated during an explosion. Accordingly, their history coincides with that of explosives. Hence, it is intriguing that, after more than 1000 years of using explosives, our understanding of the pathological consequences of blast and body/brain interactions is extremely limited. Postconflict recovery mechanisms seemingly include the suppression of painful experiences, such as explosive injuries. Unfortunately, ignoring the knowledge generated by previous generations of scientists retards research progress, leading to superfluous and repetitive studies. This article summarizes clinical and experimental findings published about blast injuries and BINT following the wars of the 20th and 21th centuries. Moreover, it offers a personal view on potential factors interfering with the progress of BINT research working toward providing better diagnosis, treatment and rehabilitation for military personnel affected by blast exposure.

## Main principles of blast physics

### Explosives & their contribution to blast injuries

Since the explosive environment determines the type and severity of blast injuries, and blast-induced neurotrauma (BINT) is an integral part of a blast injury, understanding BINT is possible only if we grasp essential knowledge on explosives, blast physics and blast–body interactions.

Gunpowder and other explosives are undoubtedly among the legacies of ancient China [1]. Evidence of metal barrels used to fire lances that propelled gunpowder bombs dates as early as the Tang dynasty (circa 618–907 AD). Since then, explosive materials have undergone a considerable development process, and today’s chemical explosives can be classified as low or high explosives [2]. Low, or deflagrating, explosives, such as gunpowder are readily combustible substances, which, when set, burn and produce gas that forces a bullet or shell smoothly out of the barrel. High or detonating explosives, such as trinitrotoluene (TNT), cyclonite (RDX) or ammonium nitrate, among others, have a shattering effect when detonated. Since these materials consist of unstable molecules, their explosive decomposition produces shock waves as end products and they do not require any external source of oxygen.

From the perspective of blast injury etiology, it is important to remember that explosive reactions differ from ordinary combustion in the velocity of the reaction. While the combustion velocity of low-energy explosives is slow and may vary within a broad range depending upon the type and physical state of the explosive material, the velocity or time of reaction for high-energy explosives is fast. Thus, in general, injuries caused by high explosives will be more complex and severe as compared with low explosive induced pathologies.

Historically, the majority of weapon development efforts were focused on improving the shattering effects of high explosives to enhance explosive efficiency for propelling metal fragments and shaped charges with high velocity into the surroundings with little attention on improving the blast wave performance [3]. Thus, the emphasis was on overcoming the protective capacity of the enemy’s body and/or vehicle armor and on inflicting blunt or penetrating injuries.

Recently, nevertheless, enhanced blast explosives, such as fuel-air explosives (FAEs)/thermobaric bombs, are being more frequently used in a range of munitions. The blast waves produced by FAE weapons differ from conventional high explosives. Namely, in the vicinity of explosion, the blast waves generated by conventional high military explosives have a high peak pressure and a relatively short duration, whereas, around the FAE explosions, the peak pressures are lower than for TNT but with a significantly longer duration [3,4]. These differences in explosive and additive materials incorporated into military explosive munitions significantly influence the injurious effects of weaponry. This finding becomes obvious when the types and severity of military injuries across the 20th and 21st centuries are analyzed, showing a shift of injury types from mainly penetrating or blunt to blast.

Thus, it is safe to say that blast injuries and BINT should be assessed from a historical perspective, bearing in mind the material characteristics of explosives used in any particular conflict. For example, the blast injuries of World War I (WWI) vastly differ from those of Operation Iraqi Freedom (OIF), partly because of the significant differences in the explosive weaponry utilized.

### Blast physics & blast–body interactions

Blast is one of the products of explosion; a region of highly compressed gas expands rapidly to occupy a volume several times greater than that of the original explosive, the solid residues from the explosive or its casing added together [5]. For example, in the detonation of 1 kg TNT, the volume of resulting gas at a temperature of 2800°C is approximately 7800 l, which is 12,500-times that of the solid explosive or at normal temperature, around 735 l (i.e., 1175-times that of the solid explosive) [6].

The blast wave, in a form of a sphere of compressed and fast-expanding gases, travels faster than sound from the center of explosion, displaces and subsequently compresses an equal volume of surrounding air at high velocity [6]. This is the overpressure phase of the blast wave, which is followed by a short period of negative pressure, the so-called underpressure phase. The Friedländer pressure [7] often used to graphically illustrate these main components of a blast wave does not depict real-life scenarios. Namely, even in unobstructed, open field conditions, the blast wave reflects from the ground or from the individual’s body. The subsequent interaction between the primary and reflecting waves augments the initial pressure wave and makes it more complex. It has been estimated that the reflected pressure of a blast wave as a function of shock strength can, for an infinite shock strength, achieve eight-times that of the incident blast wave [8].

The blast wave is followed by a high-velocity, hurricane-like blast wind causing utmost destruction of its surroundings and disintegration, evisceration and traumatic amputation of body parts [9].

The seminal works from 1950s to 1970s [6,8,10–13] posited that the severity of the injuries and the extent of damage caused by a blast wave depend on five main factors [14]: the peak of the initial positive-pressure wave (e.g., the overpressure ranges from 690 to 1724 kPa, e.g., 100–250 psi, is considered potentially lethal); the duration of overpressure; the density of the medium in which the explosion occurred (air or water); the distance from the incident blast wave, namely, the intensity of the blast overpressure declines with the cubed root of the distance from the explosion (e.g., a person 3 m/10 ft from an explosion is subjected to nine-times more overpressure than a person 6 m or 20 ft away); and the degree of the blast wave’s reflection, namely, in complex environments and confined spaces, the intensity of the blast wave can be augmented between two- and nine-times due to reflection from surrounding objects or walls (e.g., victims positioned between blast and a building often suffer from injuries two- to three-times more severe than a person in an open space). Although there are many discussions about other circumstances and elements that could influence the blast effects, the importance of the above mentioned factors remains irrefutable.

The effects of explosive blasts on the body are fivefold [15–17] (Table 1): primary blast effects cause injuries (so-called, primary blast injuries) solely through interactions between the blast wave and a living body, during which a portion of the shock wave is reflected, while another part of its energy is absorbed and propagates through the body as a tissue-transmitted shock wave [18]; secondary blast effects lead to secondary blast injuries, which can be blunt or penetrating, depending on the interactions between the fragments of debris propelled by the explosion and the body (i.e., whether the fragments damage the integrity of the skull or the skin barrier); tertiary blast effects inflict tertiary blast injuries as a consequence of acceleration/deceleration of the body or part of the body [19]; quaternary blast effects include transient but intense heat of the explosion and cause quaternary blast injuries, such as flash burns [20]; and quinary blast effects include a broad variety of potentially injurious factors, such as, carbon monoxide, the ‘postdetonation environmental contaminants’ (bacteria and radiation from dirty bombs) and tissue reactions to fuel and metal residues, among other causes [21].

**Table 1.** **Simplified categorization of the blast effects.**

**Blast effects**	**Definition**	**Consequences**
Primary	Blast waves • Front of high pressure • Compresses surrounding air • Falls rapidly to negative pressure • Over the speed of sound • Speed of micro–millisecond range • Main determinant of primary blast injury	Primary blast injuries Caused solely through interactions between the blast wave and a living body

Secondary	Fragments of debris propelled by the explosion fragments	Secondary blast injuries Blunt or penetrating injuries

Tertiary	Acceleration/deceleration of the body propelled by kinetic energy released during the explosion	Tertiary blast injuries – Acceleration/deceleration – Rotational – Impact injuries

Quaternary	Intense, usually transient heat	Quaternary injuries – Flash burns

Quinary	Numerous injurious factors released during the detonation of the explosive charge (carbon monoxide, radiation from dirty bombs, biological material, such as bacteria and organic material, chemicals, etc.)	Quinary Injuries – Hypoxia/asphyxia – Poisoning – Chemical burns – Infections – Radiation sickness – Tetanus

Occasionally, especially in the case of moderate-to-severe blast injuries, the multiple blast effects interact with the body in parallel. Some literature sources call such complex injurious environment and related injuries as ‘blast plus’ [22,23].

According to Clemedson and Jönsson [24], the high explosive shock wave in air travels with supersonic speed. Such a speed is one of the characteristics of a real shock wave. They posited that when entering the body, the original shock wave changes through interactions with the heterogeneous tissue elements causing dispersion, divergence and attenuation. Subsequently, the velocity of the wave reduces so that the main part of the pulse travels with sonic or even subsonic speed. Since it does not retain the characteristics of a shock wave in the true sense of the word, the notation of ‘pressure wave’ or ‘pressure pulse’ would be more correct.

Based on his extensive experimental work [18,25–28], Clemedson postulated that the impulse of the pressure wave and/or the pressure variations along the wave’s propagation throughout the body generate tissue-specific responses [18]. The historic explanations of the pressure wave–tissue interactions suggested spalling, inertia, implosion and cavitation as the main mechanisms [6,29]. Spallation develops at the interface between two media of different densities. As the pressure wave propagates across the denser medium toward a medium of lower density, it reflects from the boundary, creates a defect (i.e., crater) in that denser medium, and spall fractures and fragments from the boundary. Inertial effects also happen at the interface of the different densities. Namely, while tissue components with the lightest density travel the fastest, denser elements lag behind. The physical movements’ differing velocities cause stretch and strain at the interfaces, and consequently, lead to displacement, deformation or rupture of tissues and organs [17,27,30]. Implosion occurs when the pressure wave passes through a liquid medium containing dissolved gas. The kinetic energy of the pressure wave compresses the gas bubbles, so that the bubbles’ pressure becomes higher than the wave’s pressure. After the passage of the pressure wave, the bubbles re-expand and burst damaging the surrounding tissue; this mechanism is often called cavitation [31]. Besides the tissue damage caused by mechanism explained above, some findings indicate that the damaging effects of the primary blast might also depend on the frequency of the shock wave. For example, it was posited that high-frequency (0.5–1.5 kHz), low-amplitude stress waves target mostly organs consisting of structures with different densities. Examples of such organs include the lungs that contain air, blood and parenchyma or the brain with multiple interfaces between fluid (blood or cerebrospinal fluid) and parenchyma. On the other hand, low-frequency (<0.5 kHz), high-amplitude shear waves have been suggested to induce tissue damage by generating local motions that overcome the tissue’s natural elasticity (e.g., at the gray–white brain matter interface) [32].

## Historical observations on blast injuries & BINT

### Blast injuries

#### Clinical findings

Benzinger, in his work published in 1950, described the blast wave as “a shot without a bullet, a slash without a sword” [29]. Benzinger’s description only echoed the historical observations Rusca referred to in his seminal paper published in 1915 [33]. In his work, he summarized the experiences and opinions of physicians and scientists of the 18th and 19th centuries, as they relate to explosive injuries. One of the 18th century beliefs was that the “air at the head and sides of the bullet is strongly compressed and that the subsequent expansion exerts a violent impact” [29,33]. Based on this and similar assumptions, in 1906, Zalewski proposed tympanic membrane rupture as the ‘most typical condition’ caused by ‘locally compressed air’ [34]. After Zalewski’s initial theory, eardrum rupture endured as the main indicator of a blast exposure [35–37]. In contrast, findings from the Operation Iraqi Freedom conflict demonstrated that tympanic membrane perforation was rare with improvised explosive devices and other explosive weaponry. Perforation had low sensitivity for predicting serious or occult primary blast injury and, as such, was not a reliable biomarker [38]. Moreover, this and other studies have also shown that the absence of eardrum perforation does not appear to exclude other serious primary blast injuries [39,40].

It is safe to say that, besides some marginal observations made earlier, the first descriptions of blast injuries as possibly unique injury paradigms originate from WWI. As Rusca mentioned [33], individuals who have been exposed to blast described their experience as “being entirely enveloped by a fast-travelling pressure field”. The signs observed in blast-exposed soldiers included: tremors and general depression followed by coarse tremor of the limbs, inability to walk, mood swings, slow and stertorous breathing, dilated or normal pupils, ‘sluggish’ reflexes, vertigo even in light cases and often unexpected death [41]. Moreover, hemoptysis, restlessness, dyspnea, facial convulsion, diplopia and abdominal pain have also been reported in people exposed to blast. These symptoms have also been observed by others during WWII [42].

Postmortem examinations revealed edematous lungs with hemorrhage in different stages from petechiae through ecchymoses to confluent hemorrhage [41]. The junction between the alveolar tissue and the vascular and bronchial trees has been identified as the most vulnerable part of the lung since, as Rössle later explained [10], this layer “forms the transition from the pulmonary air cushion to the rigid bronchi and blood vessels”. He also added that the blast-induced lung injuries seem to be specific: since the force interacting with the chest is evenly distributed, the lesions are symmetrical, unless the position of the body was unilateral.

The rare WWI postmortem assessments noted enlarged and/or dilated heart with occasional hemorrhage in the subserosal layer of myocardium [41]. In addition, some literature sources after WWII described extensive superficial hemorrhages of the epicardium [10] as well as capsular hemorrhages in liver, spleen and kidneys [43]. Injuries have also been seen in gas-containing organs of the GI tract, such as the intestines and stomach.

#### Experimental research

Prompted by his observations during the Balkan Wars and later in WWI, the Swiss physician Rusca performed experimental research to mimic the conditions in trenches. He used rabbits exposed to nearby explosions, either in open or closed pits dug into the ground [33]. With a similar goal, Mairet and Durante [44] used experimental animals either laid on the ground or suspended in wide-meshed cages and then exposed them to blast generated by explosive charges (cheddite or melinite). Somewhat later, Hooker used muzzle flash of guns to test the effects of blast in frogs, cats and dogs [45]. He emphasized that the ‘primary shock’ causes the fatal effect and pulmonary injuries are not the main cause of the blast-induced mortality. Moreover, Hooker rejected suggestions that air emboli are caused by decompression during the ‘suction phase’ of the blast wind, and histamine and carbon monoxide poisoning from the injured lungs play significant roles in blast fatality.

The experiments performed by Krohn, Whitteridge and Zuckerman [46,47] in the 1940s used a similar experimental setup. The blast was generated by detonating 70 lbs of an unnamed explosive in paper containers placed on the ground, and the animals were raised above the ground either mounted on a scaffold or suspended from it. They ensured that none of the animals were displaced during the explosion. Desaga [11] used cylindrical explosive charges containing 60% TNT and 40% ammonium nitrate to generate blast, and the exposed animals were either in reclining or standing position. Those in a reclining position were laying on their right sides on the ground with their backs toward to blast.

Aiming to identify the most important mechanisms of the blast–body interactions, Benzinger [29] used five different experimental settings: the dog’s head exposed while its body was inside a box; the entire body of the dog was inside a box; the dog’s head was in a box, while its body was in the open air exposed to blast; (4) the dog was suspended from a scaffold with its body in the air and head immersed in water (exposed to underwater blast); and the animal’s body immersed in water with its head above the water; among others. Using a comprehensive set of outcome measures that included ECG, EEG, behavioral and neurological tests, postmortem analysis and histology, Benzinger concluded that the presence of air within or near an organ is a decisive factor for its vulnerability to blast.

The works of Shardin [6], Rössle [10], Benzinger [29] and Desaga [11], published in the German Aviation Medicine World War II in 1950, provided a significant impetus for experimental blast research. However, interestingly, in an attempt to better control the conditions of exposure, the research studies performed during the period of 1950–1980, changed their approach to the experimental design: instead of mimicking the ‘war environment’, they shifted to ‘artificial’ but more controllable technological solutions [8]. To avoid variations in peak pressures due to fluctuations in charge performances, compressed gas operated shock tubes have been designed and used with increasing frequency in experimental studies [48,49].

The work of Clemedson and the Albuquerque research team deserves a special mention. Clemedson was a Swedish physician and scientist working at the Defence Research Establishment. His experiments were essential in understanding the shock wave–body interactions, the pressure wave’s propagation throughout the body and the consequences of that passage through multiple organs [25–27]. The Albuquerque group, Bowen, Damon, Phillips, Richmond, White, Yelverton and Young, and others, was crucial for establishing the relationship between selected blast parameters and biological responses in humans and 12 animal (mammalian) species [12,13,50–52]. They developed a lethality curve (so-called ‘Bowen curve’) based on the relationship between the peak overpressure and mortality rate for these species and humans, and explained the body size/mass-dependent variances in blast tolerance with differences in lung density. This tolerance curve is still in use in the majority of experimental studies, although the pathoanatomical substrate of blast injuries in humans shifted from ‘blast’ lung to BINT. Since the ‘Bowen curve’ relies on mortality as well as acute lung injury rates, its use in the BINT research is limited and in some cases might lead to incorrect conclusions. Although body size and mass have been confirmed to influence blast tolerance, other elements, such as body geometry and biological features, should be also taken into account.

### Blast-induced neurotrauma

#### Clinical findings

##### Symptoms

In their 1946 article, Aita and Kerman [53], out of 400 cases of head injury, analyzed 34 cases in which blast exposure was the most probable and causal injury factor. The clinical picture they detailed has been repeatedly observed in later armed conflicts. Soldiers with ‘blast syndrome’ reported being stunned and losing consciousness due to blast. Following the exposure, the majority (∼65%) experienced headaches, tinnitus, deafness, dizziness and backache as well as poor memory, tension or dullness and apathy. Compared with patients with blunt, impact, nonblast traumatic brain injury (TBI), the BINT patients were more often irritated by sudden loud noise and/or crowd, and experienced emotional and physical exhaustion.

##### Postmortem findings

Mott’s article published in 1917 [41] was among the first postmortem reports describing in detail the pathological changes in the brain caused by blast. His findings included: congested veins both in the meninges and in the substance of the gray and white matter; enlarged perivascular spaces around arterioles, capillaries and venules as well as broadened perineural spaces. Mott found generalized chromatolytic changes of various intensities in the cells of the CNS, mostly affecting small cells in the pons and medulla. In the larger cells, he noted the Nissl granules were smaller and “not packed so closely together as normal”. He concluded that the overall CNS changes indicated a “relative degree of exhaustion of the kinetoplasm”.

Also in 1917, the German pathologist, von Hansemann described perforations of the ethmoid bone’s cribriform plate (i.e., lamina cribriformis), softening of the adjacent olfactory and frontal lobes, and ‘faveolate porencephalic and cortical sclerosis’ in soldiers manifesting cerebral symptoms after being exposed to blast [54]. Although these findings were later questioned as being the consequences of impact or penetrating injury mechanisms and not specifically by overpressure [10], the finding of a ‘faveolate porencephaly’, of which the main pathological substrates include cysts and cavities in the brain caused not only by destructive or cystic brain lesions, abnormal development, inflammation or hemorrhage [55,56] but also by direct damage [57], among many others, is quite intriguing. This is especially important when considering the increasing popularity of hypotheses suggesting cavitation as one of the key mechanisms underlying BINT [58–60].

In 1943, Ashcroft [61] published a case study describing a soldier injured by the explosion of a small grenade. After 2 h, his pulse was shallow and rapid, with cold and pale skin. Although he was conscious, he was apathetic and slow in replying to questions; nevertheless, his replies were deliberate and rational. Despite intensive resuscitation, he died 37 h after the exposure. The postmortem assessment showed extensive lung hemorrhage, diffuse ecchymoses in trachea and bronchi, small hemorrhages of the pericardium as well as the endocardium and myocardium of the right ventricle. The skull and the meninges were intact. The findings of the distinctly flattened gyri, tentorial grooving and a small cerebellar cone were explained as signs of increased intracranial pressure. In the parietal lobes and in the pre- and postcentral gyri, the brain was swollen and soft. On section, a clear color demarcation was observed between the white and gray matter. The histology assessment showed numerous capillary hemorrhages in the gray matter and occasional petechiae in the white matter of cerebral cortex.

In 1941, analyzing the cases described earlier by Mott and others as well as the observations of a pheasant found 90 ft from the edge of a bomb crater 12 h after two large bombs, Stewart *et al.* [62] hypothesized that the blast-induced ‘cerebral lesions’ were due to “the hydraulic-like pressure on the central nervous system in its firm encasement” caused by a “sudden compression of the thoracic cage with consequent violent back-pressure on the venous side”. Stewart’s assumption is one of the earliest hypotheses suggesting that BINT is caused not by energy transmission through the skull, but via mechanisms linked to the pressure wave propagation through the body.

##### Shell shock

Because of the lack of clinical alterations measurable by diagnostic methods of that time, the majority of published literature concluded that although BINT has some ‘organic basis’, in the long term, it is a psychological disorder rather than a distinct neurological condition [53,63]. Nevertheless, since the combination of organic changes in the brain and mental alterations observed in soldiers with blast have not been seen previously, considerable efforts have been made to provide a ‘sensible’ explanation for them. In the report published in 1915 and then, in a follow-up article published in 1919, Myers, an English physician who worked as a psychologist and held a rank of a Lieutenant-Colonel in the British Army Medical Corps during WWI, came up with a term ‘shell shock’ and ‘shell shocked’ [64,65]. Myers described three soldiers [64], who, according to his opinion, had been exposed to similar injurious environment and manifested comparable symptoms: injured by shells exploding in their vicinity; reported sleep problems before their injuries; experienced memory impairments after their injuries; and their vision, smell and taste have been affected. It is worthwhile to cite Myers’ description of the third soldier:

“A healthy-looking man, well-nourished, but obviously in extreme nervous condition. He complains that the slightest noise makes him start. His legs feel weak and he has pain in the precordial region. His sight has been very much impaired since the shock… He has slept very little the last two nights. Hands tremulous. Knee jerks normal, but the first attempts to evoke them provoked a spasm of the calf muscles and a few general convulsive movements as the patient lay in bed. His hands became very tremulous and his forehead sweated profusely. He appeared as if about to faint and says that he felt cold and dizzy, and experienced round and round movements of the stomach.” [64]

Entirely dismissing the symptoms, such as amnesia and impairments of vision and smell that pointed clearly toward organic brain damage, Myers concluded: *“Comment on these cases seems superfluous. They appear to constitute a definite class among others arising from the effects of shell-shock. The relation of these cases to those of ‘hysteria’ appears fairly certain.”* In his 1919 article [65], Myers further developed his theory about shell shock as a type of neurosis, hypothesizing that shell shock also depends on personality traits. Thus, some individuals are more susceptible to develop shell shock after being exposed to blast than the others:

“In my early experience of shell shock I came to lay great stress on disturbances of personality, and I regarded the amnesia and the bodily disorder, mutism, tremor, incoordination, spasmodic movement, so commonly observed in cases seen soon after their onset, as the expression of this change of personality, due, like it, to some functional dissociation.”

In his book published 1919 [63], Southard, an American neuropsychiatrist and neuropathologist, followed Myers’s line of reasoning to explain 589 case histories from the war literature reported between 1914 and 1918. To ascertain whether shell shock is of organic origin or ‘functional’, Southard classified the cases into the following 11 main groups: syphilopsychoses, hypophrenoses (feeble-mindedness), epileptoses, pharmacopsychoses (alcohol or drug/morphine abuse), encephalopsychoses (cases of focal brain lesions), somatopsychoses, geriopsychoses (senile impairments), schizophrenoses, cyclothymoses, psychoneuroses and psychopathoses. Having roughly differentiated the shell-shock neuroses from syphilis, epilepsy and somatic diseases, Southard concluded that the statistical majority of the cases “remains essentially functional”. Nevertheless, Southard cautioned about several issues he noticed: that “the absence of external injury is no guarantee against the existence of internal injury; cases are frequent enough in which organic and functional phenomena are combined; and the shell-shock neuroses proved to be farther away from a more functional of the psychoses than from certain organic psychosis.” These comments well illustrate Southard’s hesitation to irrefutably label shell shock as neurosis without any organic component.

From the 21st century perspective, it is curious how easily Myers dismissed the probability of TBI despite the consistent finding of the three major hallmarks of TBI (loss of consciousness, amnesia and sensory impairments). Although his ignorance about the biomechanical forces causing BINT is understandable, his teaching, which substituted searching the truth about the causes of a neurological phenomenon with forced psychiatric pretexts, irreparably slowed down the clinical research on BINT. This, in turn, led to unprepared new generations of military healthcare providers struggling to understand the long-term neurological consequences of blast exposure.

It is noteworthy that some contemporary clinicians’ and researchers’ opinions about BINT are well aligned with Myers’s position. Although Myers opted for the assumption that BINT is a type of neurosis (i.e., shell shock), a cluster of today’s researchers claim that the majority of neurological deficits seen in soldiers exposed to blast can be explained by post-traumatic stress disorder (PTSD) [66,67].

#### Experimental research

The WWI experience and analysis of clinical cases of blast injuries with experimental findings provided a firm basis for the blast researchers in 1940s. Stewart’s hypothesis about the ‘hydraulic-like pressure’ that travels through the body and reaches the CNS [62] was further analyzed by German scientist, Benzinger [29]. Summarizing his numerous experiments, he concluded that the “frequent severe cerebral symptoms after blast appear independent of whether or not the head is exposed”. Although this observation points toward the essential role of the pressure wave’s trans-corporal transmission, interestingly, this line of experimental research has not been followed. The teaching positing that “the brain may undergo direct injury such as cerebral contusion (coup–counter-coup) only as a consequence of secondary and/or tertiary blast or indirect injury such as cerebral infarction caused by air emboli” [10] and that “the brain protected by the skull is less susceptible to blast than other organs” [68] has gained wide acceptance and eventually became a dogma.

Parallel with the German and American scientists, Clemedson performed meticulously planned experiments in either open field conditions or using a pentaerythritol tetranitrate driven blast tube. He used the latest technology of the 1950s to instrument his experimental animals and gain insight into biological response mechanisms prompted by blast. In one of his well-known experiments [26], rabbits were exposed to high explosive shock waves generated in the blast tube. The pressure wave propagation in the brain was recorded using a barium titanate pressure transducer implanted into the brain through the animal’s left eye. In order to detect any possible different ways of the pressure wave’s passage to the brain, the animals were exposed in the following three different ways: with the whole body exposed to the shock wave; the whole body except the head protected from the shock wave; and only the head protected. Protection was accomplished by inclusion of the part of the body to be protected in a metal cylinder with thick walls. To ensure the best possible protection, the cylinder was surrounded by a layer of a cotton wool at least 10-cm thick, and outside this layer sheets of foam rubber were fixed. The results showed that the shock waves kinetic energy can be transmitted both through the skull and the body. Moreover, the experiments have given evidence for a certain degree of pressure transmission from the head down through the spinal column but not in reverse direction (through the spinal column to the brain). Additionally, in his experiments published in 1957 [69], Clemedson demonstrated the importance of vago–vagal reflexes and the autonomous nervous system (ANS) response to blast. Namely, the pressure wave propagation has been shown to cause apnea or bradypnea as well as hypotension and reduced heart rate. Clemedson studied the changes in respiration and heart rate in rabbits exposed to high explosive shock waves in a blast tube after bilateral cervical vagotomy or after pulmonary vagal denervation with the innervation of the sinoaortic region and heart left intact. The rapid shallow breathing that developed after the detonation in nondenervated animals was almost completely absent after cervical vagotomy or pulmonary vagal denervation. In the denervated animals, especially in the pulmonary vagally denervated ones, apnea was rare or of only very short duration. Bradycardia was also prevented by bilateral cervical vagotomy.

Interestingly, the research of the Albuquerque group and later the interest of the team of scientists at the Walter Reed Institute of Research mainly focused on blast-induced lung injuries or occasionally on the injuries of the GI tract, auditory or visual systems [70–75].

## Pathobiology of BINT

After the shock wave interacts with the body and head, a pressure wave passes through the body and head inducing complex response mechanisms, which can be divided into four main groups: primary tissue damage of the brain parenchyma caused by stretch, strain and/or rupture of parenchyma and blood vessels; changes triggered by the ANS; consequences of increased vascular load; and effects of locally synthesized and released mediators modulators (so-called ‘autacoids’) and/or immune system activation.

### Primary tissue damage

The propagation of the pressure wave, induced by the shock wave–body/head interaction, causes a relative motion of tissue components that may tear the interfaces between parenchyma, blood vessels, fluid/blood and air. In lungs, alveolar rupture, thinning of alveolar septae, circumscribed subpleural, intra-alveolar and perivascular hemorrhages have been seen to develop as direct consequences of the shock wave induced primary tissue damage [76,77].

In the brain, the rupture of the bridging veins leads to the development of subarachnoid hemorrhage (SAH) [78] and the tear of the parenchymal blood vessels to the intracerebral hematomas [60,79]. Microhemorrhages, mainly localized in subcortical regions of frontal, parietal and temporal lobes, have been seen in the brain of individuals exposed to blast [80,81]. A traumatic cerebral vasospasm with distinct temporal profile has been described in patients after blast exposure: it can develop early, often within 48 h of injury and can also present later, typically 10 or more days after initial injury [82]. Usually, traumatic cerebral vasospasm is mainly stimulated by SAH. Nevertheless, a recent experimental study implied that SAH is not necessary for shock wave induced vasospasm [83]. It has been suggested that the propagation of the shock wave through the vasculature represents a single rapid mechanical insult to the blood vessels’ lining (endothelium and vascular smooth muscle); it induces hypercontractility and remodeling, indicative of vasospasm initiation [84,85].

Experimental research further contributed to a better understanding of the mechanical consequences of the shock wave–brain interactions, such as the shear–strain damages of membranes including plasma membranes, organelles and intracellular membranes [86–88]. A recent study using a quasilinear viscoelastic material model of brain tissue encompassing blast loading rates posited that a shock wave with steepening velocity could generate regions in the brain with high strain rate and strain gradient even in locations remote from the initial site of interaction. [89]. Consequently, the high spatial gradients and high rates of strain and stress could induce injury to neuronal cells [89] through activation of proteolytic enzymes, such as calpain-2, and proapoptotic enzymes, such as caspase-3 [90,91], among many others. Subsequently, the activated proteolytic and apoptotic cascades could lead to neurogeneration. Dephosphorylation of the heavy subunit of neurofilament proteins is one of possible consequences of the pressure wave–brain interactions, and has been suggested to play important role in impaired axonal transport [92,93] observed in animal models of blast.

### Response mechanisms triggered by the ANS

The pressure wave’s propagation through the body increases the pressure inside organs [30]. In the lungs, such a pressure increase causes instantaneous pulmonary hyperinflation and stretches the alveolar walls [47,94]. This, in turn, stimulates the juxtacapillary J-receptors located in the alveolar interstitium and innervated by vagal fibers [95]. The subsequent vago–vagal reflex causes rapid shallow breathing, bradycardia and hypotension, which all are frequently experienced symptoms immediately after blast exposure. In addition, pressure receptors in the wall and trabeculae of the underfilled left ventricle may activate the C-fiber afferent nerves resulting in paradoxical bradycardia, which in turn decreases the contractility of myocardium and deepens arterial hypotension. This cardiovascular decompressor (so-called Bezold–Jarisch reflex) [96] could further contribute to cerebral hypoxia [94,97] and significantly contribute to the pathobiology of BINT.

### Consequences of increased vascular load

Experimental studies showed an early pressure surge through both arterial and venous vasculatures caused by the coupling of the shock wave with the body. In a series of experiments using pigs fitted with armor (i.e., lead- and foam-lined vest that covered the torso), individual printed circuit board piezoelectronic transducers were implanted in the inferior vena cava (IVC), common carotid artery (CCA), forebrain, thalamus, lateral ventricle and hindbrain of the hemisphere ipsilateral to the blast and in the thalamus of the contralateral hemisphere [98]. The major peaks in the CCA were seen 2 ms after blast, followed by a gradually increasing pressure in the IVC that reached the peak at about 4–5 ms after the first and largest peak pressure within the CCA. Interestingly, the major pressure peaks measured by intraparenchymal and ventricular printed circuit boards occurred later, between 136 and 138 ms after blast. The authors hypothesized that both vascular (CCA and IVC) pressure responses might be caused by myocardial compression. The slight difference between them could be resulting from the different lengths of the CCA–heart and IVC–heart paths, respectively. Since the path into the CCA is shorter from the heart, the rise might be more immediate [98].

This early pressure surge through both arterial and venous vasculatures and the resulting fluid sheer stress might increase the platelet-activating factor induced neutrophil activation [99] and intensify the release of other mediators/modulators originating from endothelial cells [100,101], which in turn might contribute to the early [102] and cyclic opening of the blood–brain barrier after BINT.

The importance of the blast-induced hydrodynamic pulse through venous vasculature has been demonstrated in recently published experimental work by Simard *et al.* [103]. In rats exposed to thorax-only blast injury, the authors found perivascular, mainly perivenular, changes in the brain which are significantly consistent with neuroinflammation: upregulation of TNF-α accompanied by the finding of ED1-positive cells (macrophages or activated microglia). It has been suggested that the hydrodynamic pulse radiates through vasculature away from its site of origin, ascending easily into the vasculature of the brain via veins since there are no valves to impede pressure transmission.

The mechanisms underlying the temporal difference between vascular and parenchymal pressure response remain unclear. The difference between the speed of a shock wave propagation in fluids (blood) as compared with its speed in solid tissues (across the skull, in brain parenchyma) might represent one of the feasible causes [16,104–107]. Different animal studies using small (rats) [106,108,109] or large (pigs, nonhuman primates) [98,110] animals showed significant pressure transients in the brain parenchyma and ventricle, which could lead to tissue deformation and disruption of normal vasoactive function.

### Effects of locally synthesized & released mediators/modulators & immune system activation

Peripheral tissue or organ disruptions stimulate the synthesis and release of autacoids, in other words, biological factors, which act like local hormones in the vicinity of their synthesis. Indeed, increased concentrations of various autacoids, such as prostaglandins, leukotrienes and cytokines, have been found in the blood of blast casualties [111–113]. These biologically active compounds exert a direct effect on several stages of cellular and humoral immunity, and act as important modifiers of both the early and late phases of the immune response [114]. For example, these compounds have been reported to stimulate migration of cells to the injury site (i.e., chemotaxis) and directly or indirectly modify the turnover of T and B lymphocytes, the production or release of lymphokines as well as the activity of T-helper or T-suppressor cells [114,115]. Experimental studies showed that the pressure wave propagation through the body stimulated inflammatory cells of systemic origin that, passing the opened blood–brain barrier into the brain, contributed to neurodegenerative processes [116,117].

**Figure 1.**
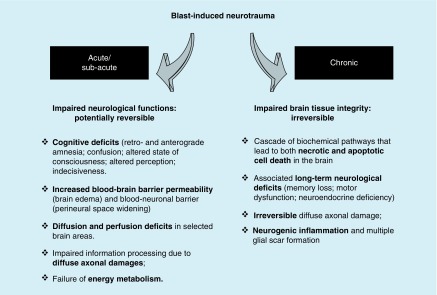
**Acute/subacute and chronic response mechanisms to blast exposure as part of the blast-induced neurotrauma pathobiology.**

The majority of these changes start as potentially reversible functional impairments which, if surpassing the counteracting control mechanisms, might lead to chronic irreversible impairments leading to impaired tissue integrity of the brain (Figure 1).

## Clinical manifestations

Recent clinical observations are very similar to those reported in earlier conflicts. Nevertheless, due to improved body armor that successfully protects from penetrating and blunt injuries (thus, from secondary and tertiary blast effects), blast injuries of the lungs and abdominal organs are less reported than in the earlier conflicts [118]. Moreover, innovations from the battlefield, including prevention of hemorrhage with the use of tourniquets and hemostatic dressings, damage control resuscitation and the rapid evacuation of casualties air evacuation teams, significantly improved the survival rates of injured military personnel [119]. These innovations, on the other hand, might translate into increased probability that the survived soldiers develop long-term consequences of their injuries. Consequently, the shift from acute injuries toward chronic ailments also changed the progress and outcome of BINT.

Patients with moderate-to-severe blast injuries and BINT often show a wide variety of multiorgan manifestations. In the lungs, various degrees of infiltration, pneumomediastinum, pneumothorax, hemothorax and pulmonary interstitial emphysema have been described, whereas in the abdomen, gastric dilation and dilated loops of bowel might be seen [76,111,112]. Clinically, these pathological changes typically manifest with dry cough, blood tinged sputum and shortness of breath. Later, tinnitus, sensorial or sensorineural hearing loss and a greater incidence of vestibular and oculomotor dysfunction, have been observed in people with BINT [112,120].

Alterations in blood biochemistry reflect the pathological changes caused by the shock wave–body/head interactions. Impaired acid–base state (decreased pH values in arterial and/or venous blood with decreased oxygen saturation) has been considered a positive finding for a possible blast injury [112], whereas UCH-L1, SBDP150 and GFAP were reported as useful tools in diagnosing BINT [121]. Our previous clinical investigations have demonstrated significant endocrine alterations as well as changes in blood total and ionized magnesium, oxidants/antioxidants and amino acid concentrations in patients after blast exposure [111,112,122–125].

BINT can cause headache, confusion, amnesia, difficulty concentrating, short-term memory loss, mood alteration, sleep disturbance, vertigo and anxiety [83,126]. Generally, these symptoms occur immediately after injury and resolve after a few hours or days. Terrio *et al.* [126], analyzing injury pattern in a brigade combat team, revealed that TBI was relatively common in that brigade combat team, the majority (88%) caused by blast. Most soldiers with BINT reported that symptoms remitted; however, 38.9% endorsed at least one mild TBI (mTBI)-related symptom at the postdeployment health assessment. While some symptoms tended to present more frequently, and resolve with time (headache, dizziness and balance problems), other symptoms were more persistent (irritability and memory problems) and nearly half of the time developed or were noted months after the acute phase. Our previous clinical studies showed unexpectedly frequent EEG changes and symptoms, such as retrograde amnesia, mental blockage, apathy/lethargy, psychomotor agitation and anxiety in blast injury patients [111,112].

### Imaging

Most recently, the steady improvement of imaging technology significantly contributed to our knowledge on BINT. The uniqueness of BINT has been well documented by imaging studies [127–129]. Patients with BINT showed greater hypometabolism on PET as compared with blunt TBI patients, especially in the right superior parietal lobe and the posterior cingulate cortex [130]. A recent study using magnetic resonance spectroscopic imaging further demonstrated the blast-induced metabolic changes in the brain [131]. The hippocampus of 25 veterans with BINT, 20 controls and 12 individuals with PTSD but without exposure to explosive blast were studied using magnetic resonance spectroscopic imaging at 7 Tesla. In the BINT group, significantly decreased N-acetyl aspartate/choline (NAA/Ch) and N-acetyl aspartate/creatine (NAA/Cr) ratios were found in the anterior portions of the hippocampus in comparison to control subjects; these changes were more pronounced in the right hippocampus, which was 15% smaller in volume than the left one. Importantly the reduction of the NAA/Ch and NAA/Cr ratios were not influenced by comorbidities, such as PTSD, depression or anxiety. The PTSD group without blast had less pronounced changes, which were mainly observed in the posterior hippocampus. The BINT patients also showed a reduction in visual memory compared with the no-blast PTSD participants. The authors concluded that the changes in hippocampal region clearly show injury dependence: they significantly differ between BINT and PTSD.

Functional MRI showed altered pattern of brain activation, specifically in the orbitofrontal–striatal inhibitory control circuit, observed during a task that involved impulse control in BINT patients more than 4 year after blast exposure [132]. Recent imaging studies imply that the caudate and striatal pathways might be specifically vulnerable to blast effects [127,132]. In their latest article [133], as a follow-up to their previous studies [23,134], MacDonald *et al.* used the method of diffusion tensor imaging (DTI) and quantitative volumetry MRI to evaluate the long-term consequences of BINT in active-duty US military, and leverage previous longitudinal data collected in these same patients to identify predictors of sustained DTI signal change indicative of chronic neurodegeneration. In total, 50 BINT and 44 combat-deployed controls were assessed at their 5-year follow-up. The analysis of the DTI findings showed that 74% of the BINT group demonstrated reductions in fractional anisotropy indicative of chronic brain injury. Logistic regression leveraging clinical and demographic data collected in the acute/subacute and 1-year follow-up identified the predictors of these long-term imaging changes. The brain injury diagnosis, older age, verbal memory and verbal fluency best predicted the presence of DTI abnormalities 5 years after injury. As the authors have emphasized, the findings provide supporting evidence for the progression of BINT toward chronic neurodegeneration even after a mild exposure, and not to its resolution.

### Postmortem findings

Traumatic encephalitis as a neurodegenerative consequence of TBI was first suggested in 1927 by Osnato and Gilberti [135]. Approximately two decades later, Critchley [136] coined the term ‘chronic traumatic encephalopathy’ (CTE), which has been widely accepted by clinicians and scientists to explain persisting clinical symptoms and underlying degenerative changes after head trauma [137–140]. Today, the term CTE is mainly used to indicate a neurodegenerative disease in individuals who suffered repeated concussions and subsequently developed progressive dementia [141,142]. The main molecular mechanisms of CTE revolve around hyperphosphorylation of τ, which leads to its dissociation from tubulin and consequent dysfunction of microtubules. These changes are followed by translocation of insoluble τ to the neuronal soma, where it accumulates forming τ oligomers. The distribution of τ-positive neurofibrillary tangles (NFTs) has been shown to be different from other tauopathies [140]. Despite the growing popularity of the hypothesis positing a link between repeated concussions and CTE, the causality of this relationship has not yet been firmly established [143].

McKee and colleagues examined 68 subjects with CTE: 64 athletes, 21 military veterans of whom 86% were also athletes and one person with self-injurious (head banging) behavior [144]. No information has been provided about the period between the last concussion and time of death (thus, postmortem analysis). The authors hypothesized that the initial CTE changes originated from perivascular foci in the sulci of cortical structures, which have increased vulnerability toward mechanical trauma. Repeated injuries, then, facilitated the spreading of those changes to the superficial layers of the lateral convexities, medial temporal lobe, diencephalon, basal ganglia, brainstem and spinal cord [145]. Nevertheless, it is noteworthy that the authors found significant comorbidities, such as Alzheimer’s disease, Lewy body disease, frontotemporal lobar degeneration and motor neuron disease. Indeed, of those cases with CTE and Alzheimer’s disease, 43% had overlapping Lewy body disease. Moreover, of those with CTE and frontotemporal lobar degeneration, 50% also had Lewy body disease. This could be, in part, caused by a relatively advanced age of the participants (mean 59.5 years, the oldest 98 years), when neurodegeneration, not provoked by concussions, might develop. Furthermore, the study did not differentiate between military personnel with and without previous history as athletes. Thus, the findings cannot be explained as military specific. The lack of data that would confirm a specific relationship between primary BINT and CTE necessitates further research including well-defined prospective clinical studies and experimental research with militarily-relevant animal models.

BINT-specific neurodegeneration has been recently reported by Shively *et al.* [146,147]. Brain specimens from five cases with chronic blast exposure, three cases with acute blast exposure, five cases with chronic impact TBI, five cases with exposure to opiates and three control cases with no known neurological disorders have been assessed. All five cases with chronic blast exposure showed prominent astroglial scarring that involved the subpial glial plate, penetrating cortical blood vessels, gray–white matter junctions and structures lining the ventricles. Moreover, all cases of acute blast exposure showed early astroglial scarring in the same brain regions. Interestingly, all cases of chronic blast exposure had a diagnosis of PTSD before death. The civilian cases, with or without history of impact TBI or a history of opiate use, did not have any astroglial scarring in the brain regions analyzed. The authors underlined that the blast exposure cases showed a distinct and previously undescribed pattern of interface astroglial scarring at boundaries between brain parenchyma and fluids, and at junctions between gray and white matter. Moreover, they pointed out that such a distinctive pattern of scarring might indicate specific areas of damage from blast exposure consistent with the previously described pressure wave–tissue interactions [17,148], and further, could account for some of the aspects of the neuropsychiatric clinical manifestations.

### Conclusion

**Figure 2.**
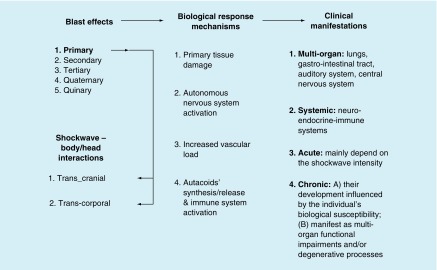
**Schematic overview of the relationships between the blast effects, biological response mechanisms and clinical manifestations as final consequences of the blast–body/head interactions.**

BINT is a consequence of the interactions between the shock wave and the body and/or head. The transcranial (through the skull) and transcorporal (through the body) couplings usually occur in parallel. The kinetic energy transferred into the brain induces complex pathological changes through local tissue responses, ANS reflex mechanisms, cerebrovascular alterations, and biochemical and molecular processes. Months and years after the blast exposure(s), the functional impairments may become chronic and trigger neurodegenerative processes as well as other long-term multiorgan deficits. Systemic alterations often play important role in chronic multiorgan dysfunctions and may include neuroendocrine insufficiency, cardiovascular instability, dyspepsia and irritable bowel, among others (Figure 2).

## Future perspective

When the carefully detailed clinical observations and excellent experimental research designed to mimic real-life scenarios during the first half of the 20th century, and the significant progress in the 21st century technology supporting clinical diagnosis and basic science are taken into account, one would expect that we fully understand BINT, know how to diagnose and treat it, and, most importantly, we have tools capable of preventing it. Unfortunately, our soldiers do not have reliable diagnostic measures, nor successful treatments or rehabilitation, and definitely, their body armor does not protect them from blast.

So, what went wrong? What has slowed down the progress in our understanding of BINT?

### Shifted experimental paradigm

While the experimental models of the first half of the 20th century have been developed to reproduce real-life scenarios, the models developed later preferred technological solutions that provided better control of the physical components of blast. Unfortunately, the conditions generated by compressed air- or gas-driven shock tubes or the blast tubes that used explosive charges not only differ widely, but they are also limited in their capacity to reproduce military-relevant environment.

The existing rigid divide between blast physicists, engineers and biomedical researchers led to numerous systems claiming to generate ‘blast’, ‘shock wave’, blast injury and BINT; unfortunately, many of them reproduce faulty conditions and/or clinically irrelevant injuries. For example, recently, several experimental devices have been described that use ultrasound [149,150] or other means of generating overpressure, such as microwave [151] or laser [152]. Nevertheless, the resulting shockwaves do not have the physical properties of a blast (i.e., an explosion-generated shockwave) and do not replicate features of BINT seen in individuals exposed to blast.

### Dichotomy between clinical reality & experimental research

The opinion voiced by some physicians and researchers between 1920s and 1940s that the neurological and behavioral impairments are functional, lacking organic origins, became a dogma during the 1960s. This doctrine explaining BINT with shell shock (later PTSD), tightly linked to personality types, familiar inheritance of psychological instability or earlier injuries, got challenged only during late 1990s, by observations emerging from military conflicts in the Balkans [111,112,125].

As a result, the clinical research stagnated, whereas the experimental research took a direction that provided a good alignment with the accepted theory about blast-induced neurological alterations: namely, that they are either caused by air emboli due to blast injury to the lungs or are a consequence of a direct head impact or head acceleration. Hence, lung injuries became the main focus of the experimental studies, while experiments aiming to assess the blast-induced changes in the brain favored a set-up that maximized the shock wave’s jet and acceleration effects. Consequently, these models reproduced impact or rotational TBIs rather than BINT [153]. Many of these models showed the neuropathology of focal brain lesions as compared with the diffuse type of pathological changes seen in patients with BINT. Accumulating clinical evidence provides growing information about the pathoanatomical substrate of BINT, its long-term progress and final outcome. The preclinical models should emulate the clinical observations in scientifically acceptable conditions, thus reproducing essential patient symptom complexes including multiorgan changes that occur simultaneously with neurological changes.

### Dichotomy between military-relevant & academic research

The current warfare is changing: from the classical form that included only conflicting military groups to urban warfare bringing dispersed armed hostility and increasing civilian casualties. The inflicted injuries and related health impairments are complex. At the same time, the military-relevant biomedical research as an academic domain is very new and extremely challenging: it has to incorporate military pragmatism, out-of-the-box thinking and scientific rigor. Besides fulfilling this three-pronged requirement, scientists interested in military-relevant biomedical research are challenged by the disconnect between the military and academia concerning priorities and problem-solving methods.

Due to the complexity of the military milieu, where multiple injurious factors often act in parallel, an out-of-the-box research approach is needed to identify the essential mechanisms underlying service-related health impairments. It has been recognized that multidisciplinary experimental models are needed to reproduce and understand the complex effects of explosion (i.e., blast) – the number one cause of injuries in modern military actions – on the human body [154,155]. The demand for such research studies is increasing since military personnel and objects ceased to be the only targets of explosive weaponry: the change of military warfare and the intensified terrorist activities made the civilian population increasingly vulnerable. There is also a dire need for longitudinal, prospective clinical studies that start at admission to military service and span across a military career: these will be essential to pinpoint the factors and their interactions in causing health impairments, and identify the signs, onset, progress and outcomes of resulting ailments.

In military-relevant biomedical research, for example, if the biological model is chosen to research BINT, then the researcher should ask him/herself how valid is that model to mimic the signs, progress and outcomes of a human BINT? What are the similarities and differences between the human body and the surrogate (animal, computational model or physical model)? What are the similarities and differences between a real-life explosion scenario and the laboratory conditions generating injury? Are the differences too large for a reliable extrapolation of the results?

Moreover, in military-relevant BINT research, the modifying effects of the environment are often underestimated or neglected. For example, without the circumstances of the blast exposure and its earliest responses, it is impossible to understand the later progression and outcome of BINT. Nevertheless, research studies are rarely conducted in a combat zone, and extrapolations are made based on the tests performed before and especially after deployment. Although the concept of a soldier living and performing in a combat zone as a ‘black-box’ [156] could be a useful philosophical exercise, its use in a research that aims at understanding the complexity of BINT is disputable. Especially, this might be the case if BINT is caused by multiple exposures to low-intensity blast and aggravated by chronic operational stressors (such as sleep deprivation, high altitude, changed nutrition, social displacement and heat or cold, among others).

**Executive summary**

- The experimental blast-induced neurotrauma (BINT) research should get closer to clinical observations and research which guarantees the optimal alignment between the BINT models and human pathology. To achieve this, a close collaboration between clinicians and biomedical researchers will be needed.
- Bearing in mind the blast physics and the features of the military-relevant injurious environment, the experimental models should reproduce operationally pertinent conditions. Moreover, providing information about the shock wave properties generated in each laboratory is essential for comparing research findings between different research groups, and confirming the clinical and military value of the data.
- To ensure the research deliverables have translational value for military personnel, a close collaboration between the military and academia is vital. Recently, recognizing the need for synchronized research efforts, several research consortia, such as the Chronic Effects of Neurotrauma Consortium [157,158] or the Understanding Neurologic Injury and Traumatic Encephalopathy [159], among others, have been established to comprehensively evaluate possible chronic and later-life effects of repeated concussions among veterans who served in recent military conflicts. Moreover, the state-of-the-art conference on traumatic brain injury and BINT organized by the United States Department of Veterans Affairs Office of Research and Development in 2015 provided an excellent platform for multidisciplinary exchange of knowledge. The workshops discussed the knowledge gained over the last several years as it relates to basic scientific methods, experimental findings, diagnosis, therapy and rehabilitation of traumatic brain injury and BINT. The state-of-the-art workshops’ recommendations are planned to provide a global framework for future research.
- Taken together, the future of the BINT research depends on how successful the research community will be in establishing a patient-/soldier-centered research strategy, where the clinical paradigm of the BINT will guide the experimental models and the feedback from experimental findings will guide novel diagnostic and treatment modalities.
